# Advancements in Particle Engineering for Inhalation Delivery of Small Molecules and Biotherapeutics

**DOI:** 10.1007/s11095-022-03363-2

**Published:** 2022-09-07

**Authors:** Rachel Yoon Kyung Chang, Hak-Kim Chan

**Affiliations:** Advanced Drug Delivery Group, Faculty of Medicine and Health, Sydney Pharmacy School, The University of Sydney, S341, Pharmacy and Bank Building A15, Sydney, New South Wales 2006 Australia

**Keywords:** biologics, dry powder formulation, inhalation, particle engineering, small molecules

## Abstract

Dry powder inhalation formulations have become increasingly popular for local and systemic delivery of small molecules and biotherapeutics. Powder formulations provide distinct advantages over liquid formulations such as elimination of cold chain due to room temperature stability, improved portability, and the potential for increasing patient adherence. To become a viable product, it is essential to develop formulations that are stable (physically, chemically and/or biologically) and inhalable over the shelf-life. Physical particulate properties such as particle size, morphology and density, as well as chemical properties can significantly impact aerosol performance of the powder. This review will cover these critical attributes that can be engineered to enhance the dispersibility of inhalation powder formulations. Challenges in particle engineering for biotherapeutics will be assessed, followed by formulation strategies for overcoming the hurdles. Finally, the review will discuss recent examples of successful dry powder biotherapeutic formulations for inhalation delivery that have been clinically assessed.

## Background

The lung has been considered as a promising drug delivery avenue in clinical practice for over a century. The pulmonary route allows fast and effective delivery of drugs for local effect and systemic uptake. For optimal delivery of drugs, the dispersibility of the formulation to form inhalable aerosols is critical. Great effort has been put into particle engineering for improving the aerosol performance of the dry powder inhalation (DPI) formulations. Particle engineering for inhalation delivery was explored in the early 1900s where ‘dry spray’ (a similar set up to what we now call ‘spray dryer’) was first designed by Körting to produce small dry particles that can reach the alveoli when inhaled [1]. Similar atomiser device was patented to produce medicines in powdered form that are suitable for inhaled delivery to the lungs [2] and several dry powder inhalers have been patented in the early 1900s [3–5]. In the 1980s, the field of particle engineering for inhalation delivery was radically improved, primarily through the works by Gonda and colleagues, who explored particle engineering for improving the powder dispersibility [6, 7] and for preventing water-induced hygroscopic growth of aerosols in the respiratory tract during delivery [8]. For example, experimental anti-cancer drugs such as hexamethylmelamine was engineered as inhalable particles for intended treatment of lung cancer [9]. Around the same time, Hickey and colleagues used lauric and capric acids to prevent hygroscopic growth of disodium fluorescein formulations in the respiratory tract [10]. Particles that are particularly prone to water absorption at high relative humidity environment can cause changes in their sizes, morphology, solid phase (amorphous to crystalline), and potentially chemical degradation, which can all impact drug pharmacology. Over the past four decades, particle engineering has continued to be explored to enhance aerosol performance of DPI formulations. More recently, pulmonary delivery of biotherapeutics such as genes, peptides, proteins, virus, and cells has become increasingly popular in respiratory medicine. DPI formulations of biotherapeutics require different formulation strategy as biologics are more labile and both biological and physical stabilities need to be considered in particle engineering.

This review will cover particle engineering strategies for improving aerosol performance and lung deposition of DPI formulations. We will discuss the influence of physical properties of the formulations such as particle size and morphology, followed by chemical engineering of particles through co-formulation with excipients or active pharmaceutical ingredients (APIs). We will then discuss challenges and formulation strategies to consider in particle engineering for inhaled biotherapeutics, and recent examples of successful cases of DPI biotherapeutics formulations.

## Engineering Physical Properties of Particles

### Size

**Fig. 1:**
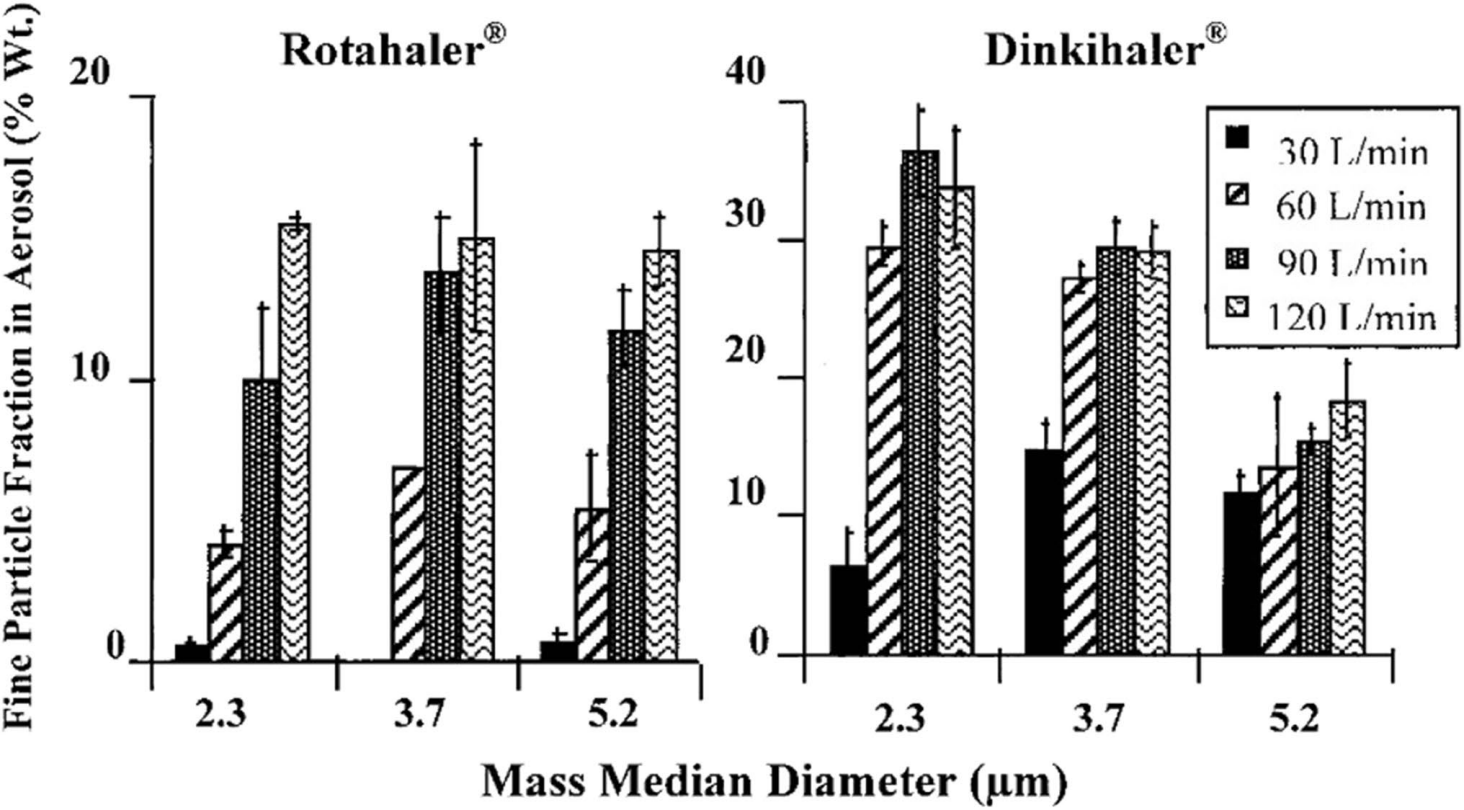
The relationship between fine particle fraction and mass median diameter of spray dried DSCG powders. Reprinted with permission from reference [15]. Copyright 2000 Elsevier.

Particle size is one of the crucial factors that influences aerosol performance, thereby impacting lung deposition and retention in different airway regions. It is expressed as the geometric diameter which reflects physical diameter of the particle, or as aerodynamic diameter, ${\mathrm{D}}_{\mathrm{a}}$. ${\mathrm{D}}_{\mathrm{a}}$ is the key parameter that determines the aerosol performance of the powder and is defined as diameter of a spherical particle with a unit density that settles at the same velocity as the particle of interest in air:

1 $${D}_{a}={D}_{g}\sqrt{\frac{1}{\chi }\bullet \frac{\rho }{{\rho }_{o}}}$$

where ${D}_{g}$ is the geometric diameter, χ the dynamic shape factor, $\uprho$ the particle density and ${\rho }_{o}$ the unit density (1 g/cm^3^). Particles with a ${\mathrm{D}}_{\mathrm{a}}$ value between 1 and 5 µm are often quoted to be desirable for lung deposition and distribution [11–13]. Generally, when the powder has a larger median particle size, the fine particle fraction (FPF, the mass % of fine particles in the aerosol < 5 µm) is low (Fig. 1). Unless being inhaled at a very low air flow (e.g., 5 L/min), particles with ${\mathrm{D}}_{\mathrm{a}}$ larger than 5 µm are likely to deposit in the oropharynx and upper respiratory tract, while those below 0.5 µm tend to be exhaled out [12]. Although it is feasible to reduce the ${\mathrm{D}}_{\mathrm{a}}$ by reducing the ${\mathrm{D}}_{\mathrm{g}}$ (see Eq. 1), particles with a small ${\mathrm{D}}_{\mathrm{g}}$ exhibit poor flowability and aerosol properties due to strong inter-particulate forces in the powder [11, 14].The cohesion between fine particles can be somewhat overcome by increasing the air force (thus, the shear force in the turbulence) through the inhaler device and the resulting impaction force between powder and inhaler interior surface. For instance, spray dried powder formulation of disodium cromoglycate (DSCG) with a mass median diameter of 2.3 µm exhibited poor aerosol performance, but was improved with increased air flow of the powder inhaler from 30 L/min to 120 L/min [15]. Similar phenomenon was observed for mannitol powders where the aerosol performance of the powder with a mass median diameter of 2.7 µm was improved by increasing the flow rate [16].

The actual *in vivo* dose depends on not just ${\mathrm{D}}_{\mathrm{a}}$, but the inspiratory flow and the complex geometry of the human upper respiratory tract (URT) which is distinctly different from the USP induction port. It has, thus, been advocated that instead of ${\mathrm{D}}_{\mathrm{a}}$ alone, the impaction parameter (which is the product of the air flow and square of ${\mathrm{D}}_{\mathrm{a}}$) is a better predictor for lung deposition [17]. Moreover, ${\mathrm{D}}_{\mathrm{a}}$ has conventionally been measured by cascade impaction with an uncoated metal USP induction port and air drawn in a square-wave profile, none of these mimic the human subject using a DPI nor captures the inter-subject variation in airway geometry or breathing pattern. Depending on the air flow, while aerosol particles of 5 µm can be captured in the human URT, they may escape the simple geometry of the USP induction port and, thus, mistaken as being inhalable. For these reasons, using 5 µm as a predictor for *in vivo* deposition has caused overestimation of lung dose while 3 µm was shown to provide a better *in vitro*-*in vivo* correlation [18, 19]. A useful and compliant approach for increasing the drug delivery to the lungs it to hold breath for 10–20 s after aerosol inhalation [20].

### Morphology

Particle shape is another property that can directly influence the aerodynamic diameter. It determines the particle packing in an agglomerate as well as the specific surface area and friction, which all influence powder flowability and emptying from a powder inhaler. The strength of the agglomerate (σ) is given by:

2 $$\sigma ={15.6\phi }^{4}\frac{W}{D}$$

where $\upphi$ is the packing fraction, *W* the non-equilibrium value of the work of adhesion, and *D* the physical diameter of the particle [21]. Hence, the agglomerate strength will be higher if the packing fraction (i.e., the volume taken by number of particles in a given volume) is high or the particles are packed more closely in an agglomerate. Particle morphology can also influence the aerodynamic diameter through the shape factor and density, where a large χ and small $\uprho$ would reduce the ${\mathrm{D}}_{\mathrm{a}}$ of a particle (from Eq. 1).

#### Elongated particles

**Fig. 2:**
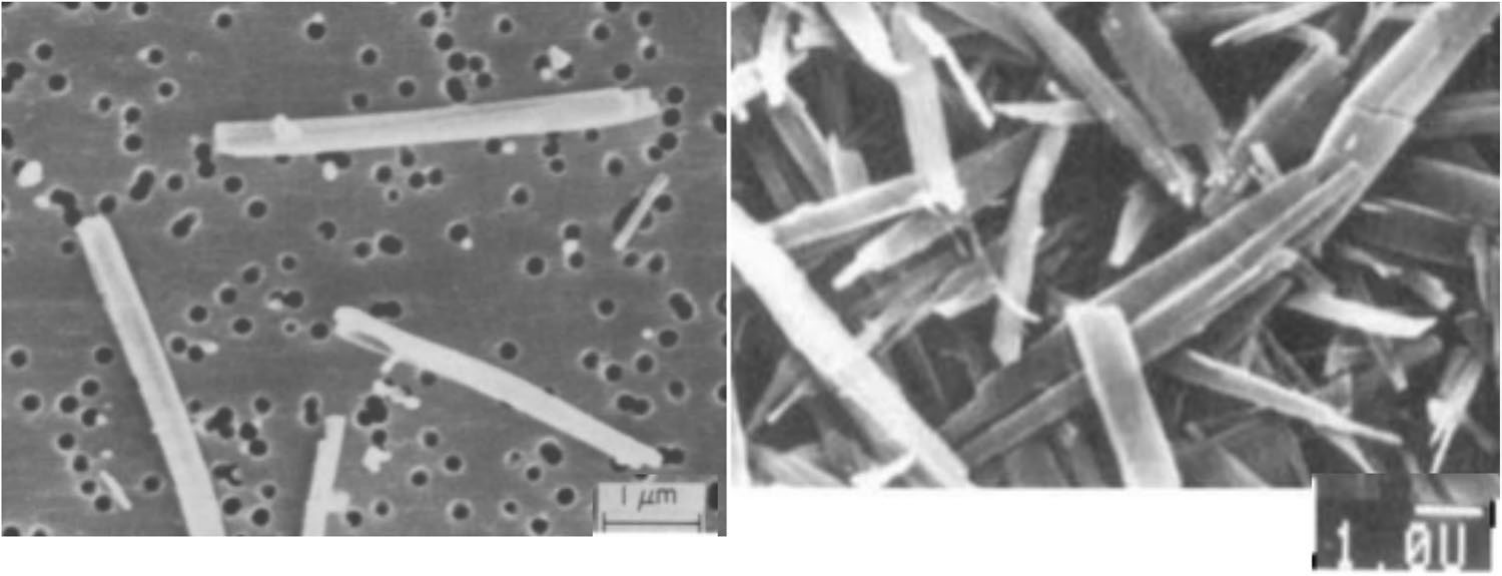
Scanning electron micrograph of cromoglycic acid (left) and nedocromil (right) particles. Reprinted with permission from references [6, 22]. Copyright 1989 & 1995 Elsevier.

Elongated particles have a larger χ value than spherical particles with the same volume or mass, which leads to a smaller ${\mathrm{D}}_{\mathrm{a}}$. The aerodynamic diameter of elongated particles is dependent on the width instead of its length [7]. Consequently, large needle-like particles that are > 10 µm in length but has a smaller width can still deposit in the lungs upon inhaled delivery. This aerodynamic advantage of elongated particles has been exploited in studies by Gonda and Chan to produce anti-asthmatic drugs cromoglycic acid [6] and nedocromil [22]. Cromoglycic acid with respective length and width values of 5 µm and 0.3 µm (Fig. 2) had superior aerosol performance and the mass median aerodynamic diameter (MMAD, i.e., the diameter which divides the population of particles by mass into 50% that are larger and 50% that are smaller than the specified value) was 0.7 µm [6]. Similarly, although the physical diameter of nedocromil was large (Fig. 2), the MMAD value was 0.9 µm [22]. Elongated particles could also have superior fluidisation and deaggregation properties as the contact area between the particles may be low depending on the packing. However, if the elongated particles become packed with their long axis tightly aligned like bricks rather than disoriented, the powder dispersibility could be compromised.

These favourable physical and aerodynamic properties of elongated particles have been further applied to inhaled biologics and antibiotics. Steroid KSR-592 was produced as elongated particles to improve respirable lung dose of the drug [23]. The needle-like crystal form of KSR-592 with dimensions of 1.8 µm in width × 41 µm in length had a FPF of 39%, while the platelike crystal form exhibited FPF value of only 5%. Needle-shaped antibiotic rifapentine was developed for inhalation delivery for intended treatment of tuberculosis infection [24]. The produced rifapentine powder had a FPF value of 83% with MMAD of 1.7 µm, reflecting superior aerosol performance of elongated particles. Elongated particles have also been shown to display minimal device-dependent dispersibility profiles. While spherical particles exhibited varying aerosol performance when dispersed by the Aeroliser and the Handihaler (FPF of 51% and 37%, respectively), the FPF values of elongated rifampicin was around 60% regardless of the device type or flow rate [25].

#### Porous Particles

Porous particles have a low particle density (< 0.5 g/cm^3^) and a large surface area (> 50 m^2^/g), which leads to a low aerodynamic diameter. These particles have lower inter-particulate cohesive force (i.e., van der Waals force) due to reduced contact area between the particles and/or reduced particle mass per unit volume of packing. As a result, porous particles exhibit less particle agglomeration and enhanced lung delivery efficiency than solid spherical particles. One of the pioneering studies has shown that large porous particles with geometric diameter of > 8 µm and particle density of < 0.1 g/cm^3^ exhibited an *in vitro* respirable fraction of 57% [26]. These porous particles could be delivered deep into the lungs of rats and escape clearance by macrophages owing to its large geometric diameter. Moreover, insulin encapsulated in porous particles exhibited higher bioavailability upon inhaled delivery to rats and suppressed systemic glucose levels for an extended period of time as compared to smaller non-porous particles (< 5 µm). Another advantage of porous particles is that lung delivery is largely independent of the peak inspiratory flow rate of patients, which entails reduced dosing variability [27, 28]. All of these traits make porous particles a desirable strategy for delivering inhalation drugs to the deep lung for treating pulmonary infections caused by bacteria and virus, pulmonary diseases (e.g., cystic fibrosis), and systemic delivery (e.g., insulin). In fact, porous particles have been exploited to enhance lung dose of antibiotics (e.g., rifampicin, tobramycin, ciprofloxacin), anti-inflammatory drugs (e.g., meloxicam, budesonide), herbs (e.g., curcumin), and biologics (e.g., genes, proteins) [27, 29–35].

Historically notable examples of porous particles are the AIR® and PulmoSphere™ technologies developed for DPI formulation in the late 1990s. For the Air technology, the drug of interest was spray dried with lung surfactant (dipalmitoylphosphatidylcholine, DPPC), albumin and disaccharides, which are GRAS (generally regarded as safe)-type excipients [36]. Due to its surface activity and low solubility in the water-based solvent system, DPPC localises at the particle surface during drying. The resulting particles have hydrophobic outer surface which can reduce the capillary forces between the particles, thereby further improving the aerosolisation properties of the powder [37]. Moreover, powders with high DPPC content can exhibit sustained release of hydrophilic drugs [38]. The AIR Insulin System contains porous particles with a geometric diameter above 5 µm and low particle density (< 0.4 g/cc). AIR Insulin exhibited comparable efficacy as the standard subcutaneously injected insulin in clinical studies [39], which may appeal to diabetes patients. Other drugs such as salbutamol sulfate [38] and levodopa [40] have also been designed using the AIR System for treating bronchoconstriction and Parkinson’s disease, respectively. PulmoSphere™ technology uses a pore forming agent (e.g., perfluorocyte bromide), surface modifier and lung surfactant (distearoylphosphatidylcholine, DSPC) to produce porous particles. Drugs can be incorporated with PulmoSphere as solution-, suspension-, and carrier-based systems. Particles produced using PulmoSphere technology are often smaller (1–5 µm in geometric diameter) and have a foam-like morphology as compared to the AIR System [41]. Drugs such as ciprofloxacin, tobramycin, budesonide, and indacaterol have been successfully incorporated in PulmoSphere particles [27, 31, 32, 42]. Due to the large size and a low drug mass-to-volume ratio of these porous particles, the total mass of the powder that can be loaded into a capsule is smaller than for powders with denser particles. Hence, for drugs requiring a high lung dose such as antibiotics, multiple capsules and inhalations may be needed to achieve a sufficient dose. Moreover, large porous particles take up larger storage volumes, which challenges the practicality of large-scale manufacture and storage, limiting their industrialised use.

#### Spiky Particles

Spiky particles have a spherical core with conical protrusions on the surface, which leads to larger geometric diameter but lower particle density. Similar to porous particles, spiky particles have shown to exhibit better flowability and aerosolisation as compared to particles with spherical particles with similar volumes and equivalent geometric diameters [43]. Conical protrusions on the particle surface increase the distance between interacting particles, thereby minimising inter-particulate cohesive forces and particle aggregation. When particles with similar size range but different morphology (sphere, plate, cube and elongated) was aerosolised, higher emitted dose and FPF were observed for spiky, pollen-like particles [44].

#### Other Morphologies

Development of particle replication in non-wetting templates (PRINT) technology has taken the morphological particle engineering to a newer level. Using a mold, particles with various shapes can be produced with precision with high batch to batch reproducibility and dose uniformity [45]. This technology has been utilised to produce pollen-like triangular shaped particles containing immunoglobulin G and lactose, cylindrical particles containing BSA and lysozyme, and torus particles containing itraconazole (anti-fungal drug), zanamivir (influenza drug), DNase, or siRNA [46, 47]. Cylindrical particles containing proteins BSA and lysozymes (geometric diameter of 1 µm) had MMAD of < 2 µm and FPF of 79% and 85%, respectively [47]. A DPI formulation of treprostinil produced by PRINT technology is currently under clinical investigation for the treatment of pulmonary arterial hypertension [48].

## Engineering Chemical Properties

### Co-Formulation with Excipients

**Fig. 3:**
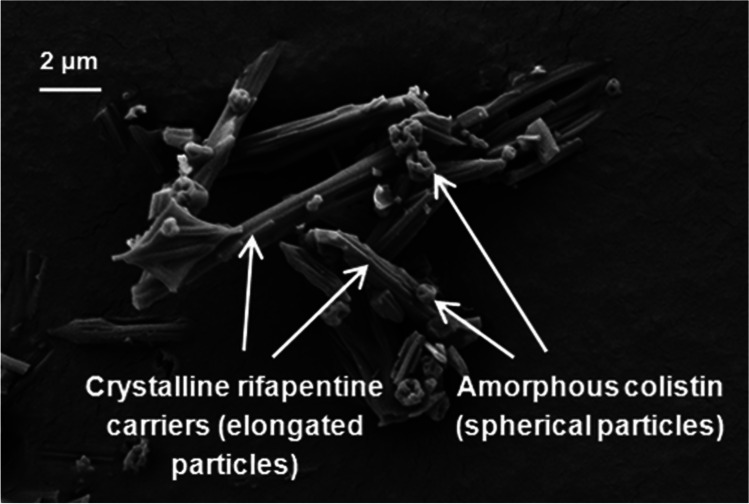
SEM images of co-spray dried colistin and rifapentine. Reprinted with permission from reference [67]. Copyright 2015 American Chemical Society.

Hydrophobic amino acids (e.g., leucine, methionine, tryptophan) are commonly used excipients for improving the physical stability and powder dispersibility of spray dried powder formulations for inhalation. L-leucine has been the most popular choice due to its surface-active and hydrophobic properties that substantially enhance the aerosol performance of spray dried powders by altering the surface morphology and surface energy of the particles [49–54]. When increasing concentrations of L-leucine (2–40%, w/w) was used to spray dry hygroscopic DSCG powders, the aerosol performance showed an upward trend with greater particle deposition in the lower stages of the impactor [51]. Compared with spray dried DSCG alone powder, those containing 2% (w/w) L-leucine exhibited significantly improved FPF_recovered_ value (72% *vs.* 58%), but the effect plateaued at leucine concentrations beyond 10–20% (w/w). The X-ray photoelectron spectroscopy (XPS) confirmed that the maximum surface enrichment was reached in spray dried powders containing 10–20% (w/w) L-leucine (i.e., 30–50 molar percent) (Fig. 3) which may have led to no further changes in the surface energies, and/or cohesive forces of the particles. Higher leucine concentration generally leads to higher powder dispersibility even after exposure to high RH [51, 55]. After 30 min exposure to 25°C/90% RH, spray dried trehalose formulation showed a significant reduction in the emitted dose (from > 90% down to < 70%) when dispersed at 60 L/min [55]. However, the presence of leucine (30%, w/w) substantially minimised the powder degradation and achieved 90% emitted dose even after 60 min of exposure to 25°C/90% RH. In another study,, the aerosol performance of the pure DSCG spray dried powders was drastically reduced (FPF_recovered_ of 2%) after one-day storage at 25°C/75% RH, which was caused by powder agglomeration resulting from strong capillary forces [51]. In contrast, DSCG powders containing 10–20% (w/w) L-leucine remained dispersible with no significant changes in the FPFs, demonstrating the protective role of L-leucine in preventing the deleterious effect of high RH on aerosol performance.

In another study, both L-leucine and L-isoleucine mitigated moisture-induced powder degradation and enhanced aerosol performance of spray dried trehalose powders [53]. The presence of L-leucine or L-isoleucine at 20–60% (w/w) improved the FPF_recovered_ of the spray dried formulations from 35% (trehalose only) to > 50%. During a 28-day storage at 25°C/50% RH, 40% (w/w) L-leucine was needed to prevent recrystallisation of amorphous trehalose, while only 20% (w/w) isoleucine was sufficient in protecting the powders. This may be due to greater surface enrichment of L-isoleucine than L-leucine when spray dried at 40–60% (w/w) which was confirmed by XPS data. Hydrophobic amino acids such as L-leucine and L-isoleucine provide moisture protection by forming a crystalline shell on the particle surface and a higher content of these amino acids often leads to greater protection [51, 53, 56]. Due to their hydrophobicity and surface activity, L-leucine and L-isoleucine become enriched on the particle surface during spray drying, followed by supersaturation and then precipitation on the surface during drying. For L-leucine to act as a physical barrier to surrounding moisture, it must exist in the crystalline form that has low water uptake propensity as compared with amorphous form, and reduce inter-particulate interaction caused by water adsorption. Organic solvents can be considered for promoting L-leucine crystallisation and enhancing surface coverage on the particle surface by modifying supersaturation level in drying droplets [57, 58]. Other hydrophobic amino acids such as trileucine, methionine, tryptophan and valine can also help alleviate the moisture-induced powder degradation [59–61]. More recently, hydrophobic D-amino acids such as D-methionine and D-tryptophan have been explored in spray dried ciprofloxacin formulations for dual benefits of moisture protection and anti-biofilm effect [61].

Metal stearates have also been widely assessed for coating the inhalable particles through spray drying or dry coating for moisture protection and aerosol performance. Magnesium stearate is recognised as safe for inhalation and approved for DPI products such as Foradil Certihaler, Incruse Ellipta, and Seebri Breezhaler. This hydrophobic lubricant can help deagglomerate co-milled API-lactose-magnesium stearate blends and improve aerosol performance, whilst protecting the formulation from high RH of 75% for 15 days [62]. Despite its excellent properties in DPI products, its use has been limited to dry coating [63–65] due to poor solubility in water and organic solvents. In comparison, sodium stearate has a relatively higher solubility in water and organic solvents and thus been co-spray dried with hygroscopic drugs to enhance aerosol performance. Compared with spray dried DSCG powder, those containing 10% (w/w) sodium stearate exhibited higher FPF value (89% *vs*. 68%) [66]. Although these hydrophobic excipients provide tremendous benefits in powder dispersibility, excessive use will likely impede dissolution of APIs and impact bioavailability in the lungs.

### Co-Formulation with Active Pharmaceutical Ingredients

Two or more APIs can be co-formulated to exploit the benefits of combination formulations. For example, the use of more hydrophobic drug can confer moisture protection and prevent loss of powder dispersibility when exposed to high RH. To achieve this, hydrophilic colistin has been co-spray dried with hydrophobic drugs including rifapentine (Fig. 3) [67], rifampicin [68] and azithromycin [69], which protected the powders from moisture-induced degradation and remained highly dispersible even after storage or dispersion at 75% RH. Moreover, the combination formulations displayed synergistic antibacterial effect. In another study, colistin was co-spray dried with ciprofloxacin, which is known to form amorphous and unstable particles once spray dried [70]. The presence of colistin positively influenced the powder dispersibility and provided moisture protection. While ciprofloxacin only powder recrystallised at 55% RH within 1 h, the co-formulation remained stable and dispersibility even after storage at 55% RH for 60 days. Similarly, amorphous and unstable spray dried kanamycin could remain stable and dispersible when co-spray dried with hydrophobic rifampicin even after one month storage at 53% RH [71]. While moisture protection resulting from surface enrichment of hydrophobic APIs can benefit physical properties of the co-formulation, excessive surface coverage may impact drug dissolution. In another study, antibiotics including azithromycin and tobramycin was co-spray dried with N-acetylcysteine which is a mucolytic agent recommended for co-administration in treating cystic fibrosis patients [72]. The co-formulations showed high FPF values (67%-98%) and remained stable after six weeks of storage at 65°C (RH unreported) and, thus, expected to remain stable at room temperature by extrapolation. Importantly, the co-formulations exhibited synergistic inhibition of *Pseudomonas aeruginosa* biofilm formulation, which impels a strong drive for producing combination formulations containing two or more APIs.

## Challenges in Particle Engineering for Biotherapeutics

Compared to small molecules, biologics are more sensitive to various stresses of powder production processes. For example, biologics are exposed to heat, mechanical shear, air–liquid interface, and drying stresses during spray drying that can cause degradation and loss of function. Similarly, the stresses of freezing, drying, mechanical shear, and concentration-induced osmotic shock during spray-freeze drying can impact the stability of biologics. As complex large molecules, biologics have more potential sites for degradation. It may undergo aggregation, deamidation, fragmentation, hydrolysis, deglycosylation, oxidation, and disulphide bond formation or breakage [73], which may compromise their therapeutic efficacy and raise potential safety concerns. In fact, molecular aggregation is the most common mechanism of protein instability and it can reduce bioactivity and increase the risk of immunogenicity [74].

While milling is the most widely used method for producing DPI products, this approach may not suitable for some labile and fragile biologics that can easily become degraded [75]. For spray drying, commonly used formulation strategies such as the use of organic solvents or high temperature for promoting formation of crystalline leucine shell and/or efficient drying of the particles should be avoided for biotherapeutics. For heat-sensitive biologics, spray-freeze drying is the preferred method of production. However, this method is less well-established in pharmaceutical industry compared to spray drying, altough scaling up is feasible [76, 77].

Once the powder formulation of biologics is produced, they need to remain stable in solid state. Due to the common use of glassy excipients to stabilize biotherapeutical molecules (see Sect. 4 below), inhalable powder formulations of these molecules are often partially amorphous, hygroscopic and sensitive to moisture-induced powder degradation. As such, surrounding moisture and/or elevated storage temperature can render the formulation to potential powder recrystallisation, which can adversely impact the stability of the biologics as well as the dispersibility of the powder.

Once the powder is administered by a subject, biologics are subject to clearance in the airways via mucociliary clearance. The beating of cilia lining actively removes any insoluble particles or microorganisms out of the lungs and into the upper airways, which is eventually swallowed. Moreover, biologics can be cleared by alveolar macrophage uptake. Due to slower transport and absorption of larger proteins (> 40 kDa), they are more likely to be cleared by macrophages than smaller proteins and peptides (< 25 kDa) [78]. In addition, biologics can be degraded by endogenous enzymes in the lungs such as serine proteases, aminopeptidases, DNase, and RNase [79–81].

## Formulations Strategies for Particle Engineering of Biotherapeutics

### Formation of Amorphous Glassy Matrix

The use of suitable excipients is critical in formulation of inhalable dry powder biotherapeutics that are biologically stable and physically dispersible. The most widely utilised excipient is amorphous glass formers such as disaccharides, polysaccharides, and polyols (e.g., lactose, trehalose, sucrose, maltodextrin) [73, 82–85]. Proteins and peptides are immobilised, and hence stabilised, inside amorphous glass matrix. In the glassy state, the local mobility of the molecule is suppressed, which in turn slows down the molecular dynamics of the biologics incorporated inside the matrix, thereby slowing down the degradation process in the powders [26, 27]. This helps to maintain the structural integrity of the biologics in solid state over the shelf life. Hydrogen bonding between disaccharide molecules in the matrix and the biologics can further help slow the degradation. In fact, the stability of protein in solid state can be improved by increasing the sugar-to-protein ratio until sugar interacts with all the accessible hydrogen bonding sites on the protein surface [86, 87]. It has been suggested that in general a sugar:protein weight ratio of 1:1 to 1:5 is needed for preserving the structural integrity of proteins [88]. In addition to disaccharides, other glass forming excipients such as mannitol and amorphous calcium carbonate have been used for stabilising proteins [89, 90]. Notably, although mannitol is known to form crystalline particles when spray dried due to its low glass transition temperature (T_g_), it formed amorphous particles in the presence of salmon calcitonin when the excipient content was ≤ 50% (w/w) [89]. Although lactose is an amorphous glass former and a commonly used drug carrier, the use of this excipient in biologics has been limited due to its reducing nature that causes Maillard reaction with amino groups of proteins [78].

The same strategy has been utilised for stabilising nucleic acids and virus particles (bacteriophage) in inhalable powder formulations, where disaccharides (lactose, trehalose) or sugar alcohol (mannitol) are used for immobilising and stabling them in amorphous matrix whilst acting as the bulking agent [49, 91–94].

### T_g_ Manipulation

**Fig. 4:**
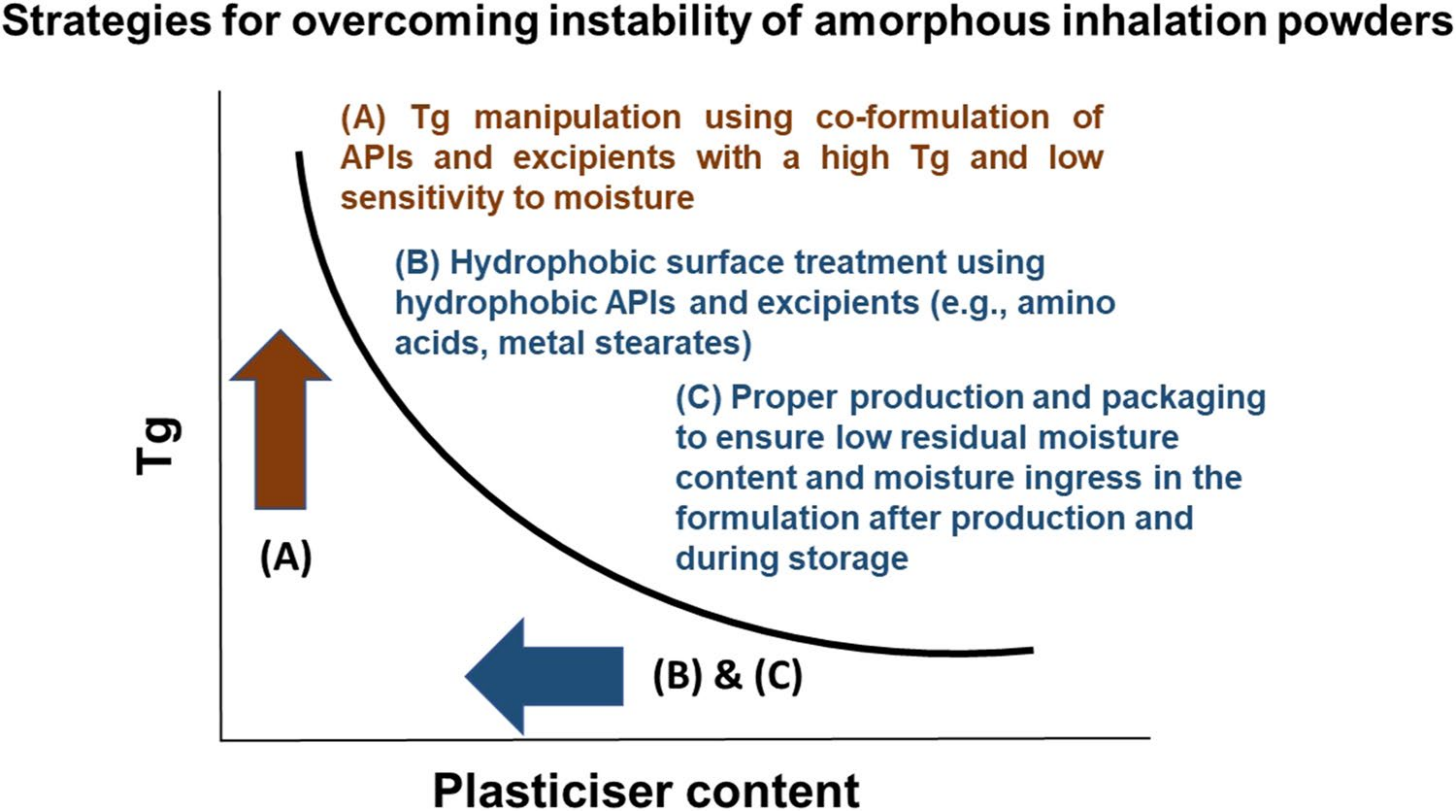
Risk mitigation strategies to overcome instability of amorphous inhalation powders. Adapted from [137].

Glassy materials are physically unstable and fine particles can quickly uptake surrounding moisture due to high surface area and high energy state. Subsequent recrystallisation is detrimental for the incorporated biologics as in the absence of amorphous matrix, they are no longer stabilised through immobilisation [95]. An amorphous glass is characterised by a T_g_ at which the matrix transitions from the glassy state to a rubbery state. As amorphous sugars are prone to recrystallisation at temperatures above T_g_ [96, 97], excipients with a high Tg can be added to increase the T_g_ of the co-formulation (Fig. 4), thereby promoting the stability of biologics in solid state. High molecular polysaccharides often possess higher T_g_ than smaller saccharides. Among disaccharides, trehalose is commonly used owing to its amorphous glass forming properties with a high T_g_ value of 120°C, while most others range between 65 and 100°C [98]. The addition of polysaccharides such as Dextran 70 kDa to trehalose can increase the T_g_ of the co-formulation and enhance the stability of proteins [99], which highlights the benefit of T_g_ manipulation in stabilising biologics in solid state. More recently, a polysaccharide pullulan has also been utilised in stabilising biologics in solid state due to its high T_g_ value of 261°C [100]. Anti-*Campylobacter* bacteriophage spray dried with pullulan and trehalose produced fully amorphous powders with biological stability during storage. Water is an excellent plasticising agent that can reduce the T_g_ of amorphous powders [101]. As T_g_ increases with decreasing moisture content [102], the powders must have sufficiently low moisture content so that the amorphous matrix remains stable and immobile in the dried state. This may be a challenge for powder stored in a capsule which can exchange its moisture with the powder to potentially its T_g_. Gelatin capsules, in particular, should be used with caution as they require certain moisture content (13–16 wt.%) to maintain its integrity being not too soft or brittle. For this reason, hydroxypropyl methylcellulose capsules which have a lower moisture content (4–6 wt.%) are increasingly used for DPI powder [103]. In addition to T_g_, the molecular flexibility of sugars can impact their ability to stabilise proteins with more flexible sugars providing better protein stability [99] probably through enhanced molecular interactions.

### Particle Surface Coating

When proteins are spray dried with excipients, the outer particle surface tends to be enriched with proteins that are surface-active, whereas small molecule excipients diffused into the core of the particle [73]. Moreover, large molecular size of proteins and macromolecules lead to slower inward diffusion from the drying interface [104]. To avoid protein degradation via unfolding or aggregation at the air–water interface, surface-active excipients such as leucine, tri-leucine, and polysorbate can be utilised [59, 84, 105–107]. These surface-active excipients can displace proteins at the interface to provide protection against protein degradation. In the same manner, surface-active excipients can minimise protein adsorption with subsequent degradation at the ice-water interface during spray-freeze drying [83, 108]. Moreover, the use of surface-active hydrophobic excipients such as leucine has additional advantage of powder dispersibility enhancement as well as moisture protection (see Sect. 2.1) (Fig. 4). However, these hydrophobic excipients often exist in crystalline state in spray dried powders, which can lead to phase separation with the biologics, causing subsequent de-stabilisation and inactivation [109]. Hence, the hydrophobic excipients must exist in amorphous state, or sugars (see Sect. 4.1) that form amorphous matrix also need to be present in the final formulation.

### Encapsulation In Micro and Nanoparticles

Polymeric nanoparticles comprising various polymers such as chitosan, phospholipid and amphiphilic polymers have also been exploited to encapsulate biologics to minimise degradation at the air–liquid interface during drying [110–113]. These polymeric particles can also promote bioavailability of the encapsulated biologics through improved transepithelial transport and reduced mucociliary clearance [112–114]. Moreover, encapsulation in polymeric or liposomal formulations can assist with intracellular delivery of biologics such as genes and antimicrobial peptides, proteins and bacteriophages [115–118]. Prud’homme and colleagues have successfully used flash nanoprecipitation techniques to fabricate a number of nanoparticles of biologics including some with encapsulation efficiency being close to 100% [119]. However, in general issues arising from the use of organic solvents in encapsulated particle production, relatively low encapsulation efficiency, and further formulation as highly dispersible inhalation powders are yet to be resolved.

### Storage and Packaging

**Fig. 5:**
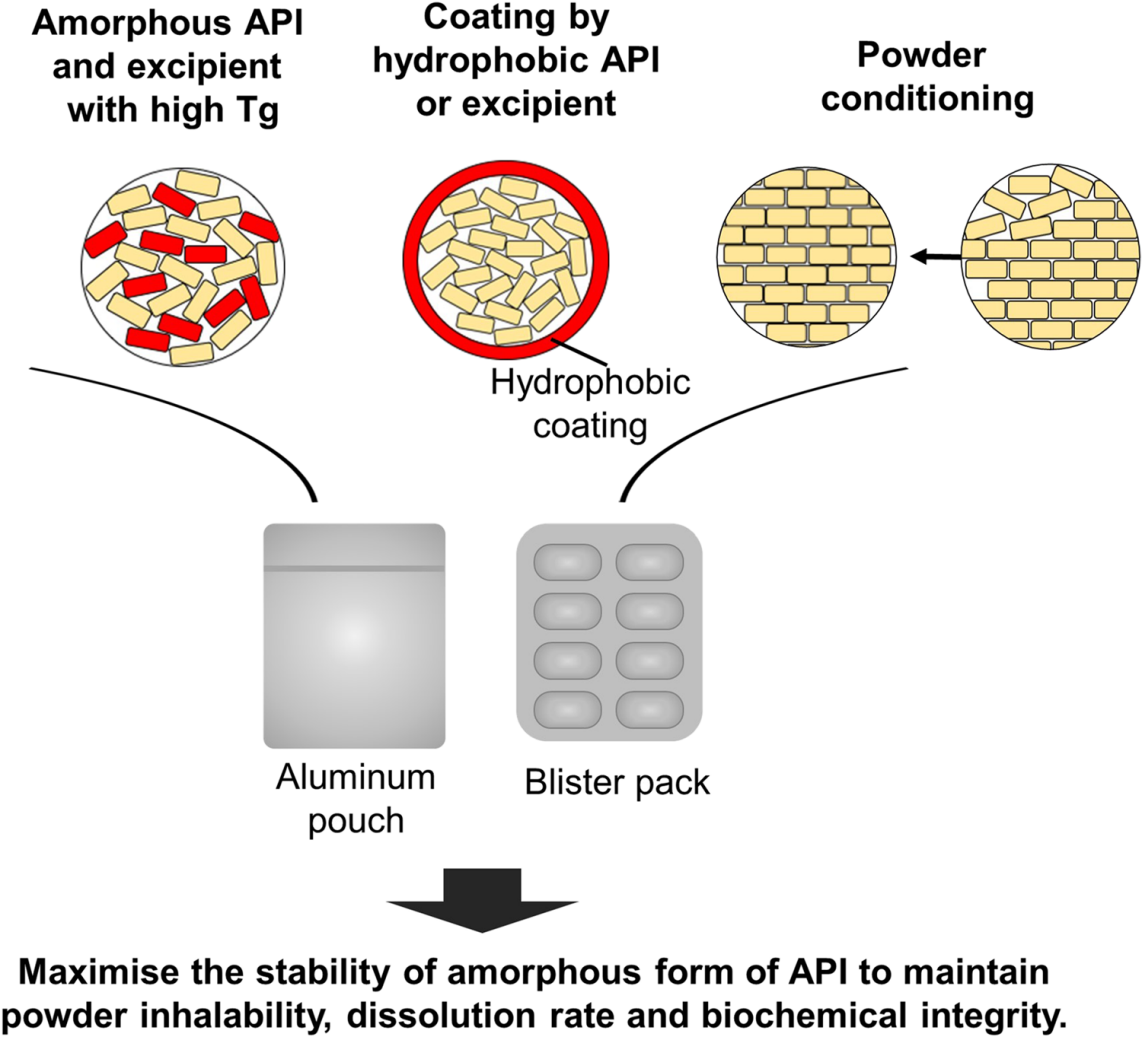
Formulation and packaging strategies for amorphous powders. Adapted from [137].

One of the key advantages of powder formulation over liquid formulation is the room temperature storage stability which negates the need for cold chain storage. To achieve ambient stability, proteins need to be stored at a temperature ~ 50°C below the T_g_, which can significantly reduce the molecular mobility [101]. As discussed in Sect. 4.2, the presence of water in the environment can decrease the T_g_ of the formulation through its plasticising effect and increase the local molecular mobility. Hence, not only the temperature but also the RH is critical in promoting the stability of biopharmaceutic powders. Similar principles also applied to spray dried powders of bacteriophage. To preserve the biological activity of this biologic, the formulation also required storage at ~ 46°C below the T_g_ [120]. When the T_g_ was manipulated through the plasticising effect of water, the bioactivity of bacteriophage reduced substantially. As such, powders can be sealed inside an aluminium pouch or blister packs at low or 0% RH (nitrogen or air gas) to eliminate moisture-induced changes to the T_g_, and hence improve the biological and physico-chemical stabilities of the biotherapeutic formulation (Fig. 5). Dry powder inhalers with discrete drug containment such as aluminum blisters should be utilised over those with a powder reservoir to reduce the risk of moisture-induced powder degradation. Capsule-based devices such as Aerolizer and Osmohaler are also suitable as individually wrapped capsules can be stored in moisture protected packages and loaded into the device and then inhaled as needed [73].

## Recent Examples of Successful DPI Biotherapeutics Formulations

Since the development of inhaled insulin powder by Inhaled Therapeutics, significant research efforts have been invested on developing DPI formulations of biotherapeutics such as peptides, proteins, monoclonal antibodies, vaccines, and virus. This section will discuss the recent examples of such biotherapeutics that have been clinically evaluated in the last three years.

### LTI-03

LTI-03 is a caveolin-1-scaffolding-protein-derived peptide being developed by Lung Therapeutics for the treatment of idiopathic pulmonary fibrosis that is characterised by progressive destruction of the lung parenchyma due to excessive formation of fibrosis. This peptide prevents excessive fibroblast growth and expansion, thereby restoring the balance in the lung and promoting protection of healthy lung epithelial cells. Both liquid and powder formulations of LTI-03 significantly reduced fibrosis and promoted epithelial cell survival in a dose-dependent manner in murine models of fibrosis [121]. LTI-03 powder formulation was prepared by air-jet milling without excipients and exhibited a MMAD value of 1.6 µm and FPF of 93% when dispersed through a Monodose RS01 high resistance dry powder inhaler [122, 123]. Recently, the safety, tolerability and pharmacokinetics of LTI-03 inhalation powder was assessed in healthy adult subjects (ClinicalTrials.gov No. NCT04233814). In this Phase 1a clinical trial, ascending doses of LTI-03 from 2.5 mg to 10 mg were well-tolerated with no reports of serious adverse events or discontinuations [124]. As LTI-03 is expected to be efficacious at doses between 1 and 10 mg in humans according to the preclinical study, further Phase II and III investigations are anticipated in the near future.

### CSJ117

CSJ117 is a neutralising antibody fragment that can help regulate asthmatic airway inflammation by targeting thymic stromal lymphopoietin (TSLP). The compound is being developed by Novartis Pharmaceuticals and is formulated as a PulmoSol™ engineered powder in capsules for aerosol delivery using a dry powder inhaler. PulmoSol technology has initially been developed for preparing spray dried insulin Exubera® by Nektar Therapeutics (previously Inhaled Therapeutics) but has now been applied to CSJ117. A recently completed clinical trial (NCT04410523) has demonstrated that a daily dose of 4 mg administered over 12 weeks was generally safe and well tolerated by mild asthmatic subjects and reduced allergen-induced bronchoconstriction [125]. Anti-TSLP agents such as CSJ117 have been recognised as a promising new therapeutic class for the treatment of asthma.

### PRS-060 (Also Known as AZD1402)

PRS-060 is an inhaled dry powder formulation of an anti-asthmatic anticalin protein engineered from endogenous lipocalin-1 that is under development by Pieris Australia and AstraZeneca for treating patients with moderate to severe asthma. In the recent Phase Ia clinical trial (NCT03921268), the safety and tolerability of PRS-060 administered by the Pastiape Monodose inhaler was assessed; formulation information was not publicly available. Following promising safety data from the study, AstraZeneca is progressing into assessing the safety of the high dose (Phase Ib) as well as efficacy of the low and medium doses (Phase IIa) in asthmatic patients [126].

### Inhaled Vaccine

Another example worth mentioning is dry powder vaccines for inhalation. Inhaled delivery of vaccine against respiratory infections is a potentially powerful strategy that can induce both systemic and local immunity in the lungs. To date, dry powder measles vaccination is the only clinical trial conducted using a DPI vaccine formulation. Live-attenuated measles virus was spray dried with myo-inositol and other stabilising excipients such as gelatin, arginine, and histidine using a modified spray drying method (i.e., carbon dioxide assisted nebulisation with a Bubble Dryer®) [127, 128]. When administered through a Puffhaler® or a Solvent™, the inhaled drug was well tolerated in all subjects [129]. Many other vaccine candidates that are currently being tested in clinical trial entails protein subunit vaccines [130]. Hence, similar formulation strategies covered in Sect. 4 can potentially be applied to produce vaccine powders for inhalation. Excipients such as inulin, mannitol, trehalose, dextran, leucine, and trileucine have been utilised to produce inhalable vaccine powders [131–136], but no further clinical studies have been conducted to date.

## Conclusion

With successful clinical translation of insulin inhalation dry powder products, a growing number of biotherapeutics are being developed as inhalable dry powders. To develop inhalation powders of biotherapeutics, various formulation strategies have been utilised in achieving biological stability and structural integrity of biologics in solid state whilst exhibiting sound aerosol performance. Indeed, the formulation strategies described in this review have been implemented by numerous published studies in developing inhalable powder formulations of biologics such as genes, peptides, proteins, virus, bacteriophages, monoclonal antibodies, and cells. Dry powder formulations of biologics heavily rely on the use of stabilising and/or bulking agents, and the limited number of approved excipients for inhalation use poses a significant challenge to the development. However, as commonly utilised excipients such as trehalose, leucine and sucrose are generally regarded safe, the safety issue is anticipated to be low although safety study is still necessary. Nonetheless, high biologics loading in powder should be attained whenever feasible as high concentrations of excipients in the lungs can potentially cause unnecessary adverse effects. Identification and subsequent FDA approval of biocompatible excipients that help stabilise biologics and enhance aerosol performance will greatly help expedite the development process. Another aspect to consider is the potential safety concerns for proteins that become denatured or aggregated during manufacture, delivery and storage. To avoid induction of immune responses by degraded proteins, physical as well as the biochemical stability needs to be monitored over the shelf-life of the formulation.

With the current COVID-19 outbreak, safe and effective vaccine is highly sought after, which has led to heightened interest in inhaled vaccine as it may provide non-invasive mucosal immunisation that trigger both local and systemic responses. The push for mRNA- and protein-based vaccine against pulmonary infections is expected to catalyse the pharmaceutical research in inhaled biotherapeutics. Yet, inhaled biotherapeutics are still at the early stages of development as compared with small molecules. Consequently, most of the ongoing clinical trials focus on the use of liquid formulations of biotherapeutics (e.g., deoxyribo-nuclease I, alpha-1 antitrypsin**,** anti-human thymic stromal lymphopoietin monoclonal antibody fragment) delivered by nebulised aerosols. Once the field of liquid formulation of biologics matures, powder formulations will naturally attract attention as the second-generation products. Currently, there is no single formulation strategy that can be applied to different types of biologics (let alone different proteins), necessitating systematic development process for individual biotherapeutics by considering the stability of the molecule in the formulation and the lungs, the use of suitable excipients and device, patient population, and any biological barriers in the lungs. For powder formulations, there have been significant progress in engineering particles for inhalation over the past three decades to enable sufficient delivery of biologics by inhalation.
